# Structural priming in sentence comprehension: A single prime is enough

**DOI:** 10.1371/journal.pone.0194959

**Published:** 2018-04-02

**Authors:** Maria Giavazzi, Sara Sambin, Ruth de Diego-Balaguer, Lorna Le Stanc, Anne-Catherine Bachoud-Lévi, Charlotte Jacquemot

**Affiliations:** 1 Département d’Etudes Cognitives, Ecole Normale Supérieure—PSL Research University, Paris, France; 2 Laboratoire de NeuroPsychologie Interventionnelle, Institut National de la Santé et Recherche Médical (INSERM) U955, Equipe 01, Créteil, France; 3 Université Paris Est, Faculté de Médecine, Créteil, France; 4 Université Libre de Bruxelles, Hôpital Erasme, Département de Neurologie, Bruxelles, Belgique; 5 ICREA, Barcelona, Spain; 6 Cognition and Brain Plasticity Unit, IDIBELL, Barcelona, Spain; 7 Department of Cognition, Development and Educational Psychology, University of Barcelona, Barcelona, Spain; 8 Institute of Neuroscience, University of Barcelona, Barcelona, Spain; 9 Centre de référence maladie de Huntington, Hôpital Henri Mondor, AP-HP, Créteil, France; Leiden University, NETHERLANDS

## Abstract

Experiencing a syntactic structure affects how we process subsequent instances of that structure. This phenomenon, called structural priming, is observed both in language production and in language comprehension. However, while abstract syntactic structures can be primed independent of lexical overlap in sentence production, evidence for structural priming in comprehension is more elusive. In addition, when structural priming in comprehension is found, it can often be accounted for in terms of participants’ explicit expectations. Participants may use the structural repetition over several sentences and build expectations, which create a priming effect. Here, we use a new experimental paradigm to investigate structural priming in sentence comprehension independent of lexical overlap and of participants’ expectations. We use an outcome dependent variable instead of commonly used online measures, which allows us to more directly compare these effects with those found in sentence production studies. We test priming effects in syntactically homogeneous and heterogeneous conditions on a sentence-picture matching task that forces participants to fully parse the sentences. We observe that, while participants learn the structural regularity in the homogeneous condition, structural priming is also found in the heterogeneous condition, in which participants do not expect any particular structure. In fact, we find that a single prime is enough to trigger priming. Our results indicate that–like in sentence production–structural priming can be observed in sentence comprehension without lexical repetition and independent of participants’ expectation.

## Introduction

Language processing is a complex process that involves–among other steps–a lexical analysis to retrieve the meanings of words and a syntactic analysis to parse the words into a syntactic structure. In sentence production, there is considerable evidence for abstract structure building. Several studies have demonstrated that the production of a syntactic structure is facilitated when this structure appears in the preceding sentence. This effect is called structural priming [1, 2]. In sentence production tasks such as sentence completion, sentence recall and picture description, participants tend to use the same abstract syntactic structure as the one to which they were previously exposed [1, 3–6]. For instance, participants are more likely to describe an event using a Prepositional-Object dative structure (PO, e.g. *“*Mary gave a bone to the dog”) if the previous description contained a PO structure. Similarly, after a Double-Object dative (DO, e.g. “Mary gave the dog a bone”), they preferentially produce sentences containing a DO structure. In the same way, participants are more likely to describe a picture using a passive structure (e.g. “John was kicked by Mary”) after being presented with a passive sentence than after being presented with an active sentence (e.g. “Mary was kicking John”) [1, 4, 7, 8]. Crucially, structural priming is observed even when the sentences do not share any lexical content, providing evidence for the idea that in sentence production the syntactic structure can be accessed and processed independent of meaning and sound [9].

The picture emerging from research on structural priming in sentence comprehension is less clear. In contrast to language production, several studies showing that comprehending a sentence with a particular syntactic structure can ease the process of comprehending a subsequent sentence with the same syntactic structure, hinge on the prime and the target sentence having the same verb [10–15]. For instance, Branigan and collaborators [16] showed no evidence for priming effects in active/passive structures and PO/DO structures when the verb was not repeated. Processing the sentence “The defendant examined by the lawyer was guilty” was speeded by “The engineer examined by the board passed with flying colors” but not affected by “The engineer tested by the board passed with flying colors” (for a review see [9, 16–18]).

The need for lexical overlap in these sentence comprehension studies may reflect the fact that, in contrast to language production, language comprehension is not completely independent of lexical processing. Indeed the difficulty for finding structural priming in sentence comprehension without lexical support may stem from differences in the way syntactic processing takes place in production and in comprehension [19–21]. Construction of a syntactic structure is mandatory for speaking: the speaker needs to select, among numerous possibilities, the syntactic construction to be used. Comprehension may differ in that word order and the sole analysis of the lexical content are often sufficient to derive the meaning of the sentence, without fully analyzing the syntactic structure. Structural priming in comprehension may rely more on lexical support because language comprehension can be successful even without full syntactic parsing [22, 23].

However, several types of results challenge this explanation. First, other studies have reported that exposure to a syntactic structure affects subsequent productions: after hearing a sentence, people tend to produce sentences with the same structure. This suggests that perceived structural representations affect language production and that structural priming can be observed both in comprehension and in production albeit possibly in different ways [3, 24–29]. Segaert and collaborators investigated the neural correlates of structural priming in sentence production and comprehension [30] (see also [31]). Whereas they identified brain regions whose activation was affected by syntactic repetition whatever the processing modality, they found no region showing differential effects for comprehension vs. production. Second, examining structural priming in comprehension and production within the same paradigm, Tooley and Bock [32] found comparable priming effects in the two modalities, even when there was no verb repetition between the prime and the target. These results rather seem to suggest that structural priming in the two modalities is a manifestation of the same, or a similar mechanism [3, 11, 33, 34].

Finally, structural priming in comprehension has also been reported in the absence of lexical repetition. Mehler and Carey [35] found that participants better understood sentences masked by white noise if they were preceded by sentences with the same syntactic structure (see also [36]). In the seminal studies by Mehler and Carey it could be argued, however, that since the prime was composed of several sentences sharing the same structure (n = 10), participants exploited the repetition of syntactic similarities between the primes and the target. More recent studies however have shown that blocked designs promoting predictive effects are not necessary conditions for structural priming to be observed in comprehension. For instance, facilitation effects have been reported in children processing double-object and prepositional-object dative sentences preceded by sentences sharing the same syntactic structure [37, 38]. These priming effects were observed independent of lexical repetition and of predictive effects, since only two prime sentences preceded the target. Similar priming effects were also found in adults. In an eye-tracking study, Traxler (2008) shows reading-time facilitation for temporally ambiguous sentences in which a prepositional phrase (e.g. “in the box” in “The vendor tossed the peanuts in the box into the crowd during the game”) could be temporarily interpreted as an argument (i.e. as in “The vendor tossed the peanuts in the box just now”) rather than an adjunct of the preceding noun (i.e. “the peanuts that were in the box”), when it was preceded by a single prime with no lexical overlap, and with the same structural analysis for the prepositional phrase [17]. Similarly, Pickering et al. (2013) observe persisting priming effects on the interpretation of prepositional phrases which were ambiguous between a high and a low attachment (i.e. between a prepositional phrase modifying the verb, e.g. The policeman is [prodding the doctor] [with the gun] and one modifying the noun, e.g. The policeman is prodding the [doctor with the gun]). Interpreting a sentence with a given interpretation primed participants’ interpretation of subsequent sentences, independently of lexical overlap, and even across intervening filler sentences [39]. Traxler and Tooley (2008) directly investigated the role of strategic effects on syntactic parsing during comprehension [40], using eye-tracking. They showed that under certain conditions structural priming in comprehension arises independent of predictive effects, like in production.

Therefore, while structural priming in speech production is a robust phenomenon attested across a wide variety of syntactic constructions and experimental paradigms, evidence for it in language comprehension is attested but less consistently observable. The dependent measures used to assess priming in the two modalities are however not the same. Production studies measure whether speakers’ exposure to a given syntactic structure affects their choice between syntactic alternatives. On the contrary, most comprehension studies examine whether syntactic priming reduces the reading time–and thus the processing difficulty–associated with a temporarily ambiguous sentence. Kim et al. (2014) assess the possible role played by these methodological differences in determining the discrepancies observed between priming in production and perception, and show that priming without lexical overlap in comprehension emerges under experimental conditions that closely approximate those used to test for syntactic priming in production [41].

This paper addresses the issue of whether structural priming in sentence comprehension can be observed in a paradigm which is comparable to those used in production studies. We investigate whether syntactic priming arises in comprehension independent of lexical repetition and of participants’ expectations using an outcome dependent response instead of an on-line processing measure.

We designed a picture-matching task with active and passive sentences, in which participants were unable to use statistical information about the syntactic structures in the environment to adapt their expectations. We forced participants to syntactically process each sentence by using reversible sentences in which neither a word-order analysis nor world knowledge could be used to comprehend the sentences accurately (e.g. active sentence: “the pineapple cuts the apple”, passive sentence: “the apple is cut by the pineapple”). Indeed, if the degree to which listeners are engaged in syntactic processing in sentence comprehension depends on the syntactic demands of the task, paradigms that do not trigger full syntactic processing ([23, 42–44]) could reduce the priming effect due to syntactic repetition. In contrast, when participants are actively engaged in sentence comprehension a syntactic structure can be extracted and reused [35].

In each trial, participants chose whether the sentence correctly described the presented picture. Dependent variables were accuracy and reaction times. We compared two conditions: a homogeneous condition in which the same syntactic structure was repeated, and a heterogeneous condition in which actives and passives were pseudo-randomly presented. In the latter condition, a constraint imposed on the randomization did not allow sequences of more than three sentences with the same syntactic structure (i.e. a sentence could be preceded by maximum two sentences with the same structure). If structural priming in comprehension relies on participants’ detection of syntactic similarities between consecutive sentences, we should only observe it in homogeneous blocks where syntactic structure is constantly repeated and can thus be expected. If on the other hand–like in sentence production–structural priming in comprehension is independent of participants’ expectations, a single structural repetition will be able to create priming in the heterogeneous condition.

## Materials and methods

### Participants

Forty-eight native speakers of French participated in the study (mean age 22.1 ± 3.7 years; 31 females, mean educational background 14.8 ± 1.4 years; all right-handed). Participants had normal or corrected-to-normal vision, normal audition, and no history of neurological or psychiatric illnesses. The study was carried out in accordance with the Declaration of Helsinki. Participants gave their written informed consent according to the French legislation. In France, behavioral, non-interventional research with healthy participants such as the one carried out in the present study lies outside of the legislation of the Huriet law concerning the protection of individuals involved in biomedical research and is thus not subject to the approval of the Ethical Committee.

### Materials

**Fig 1:**
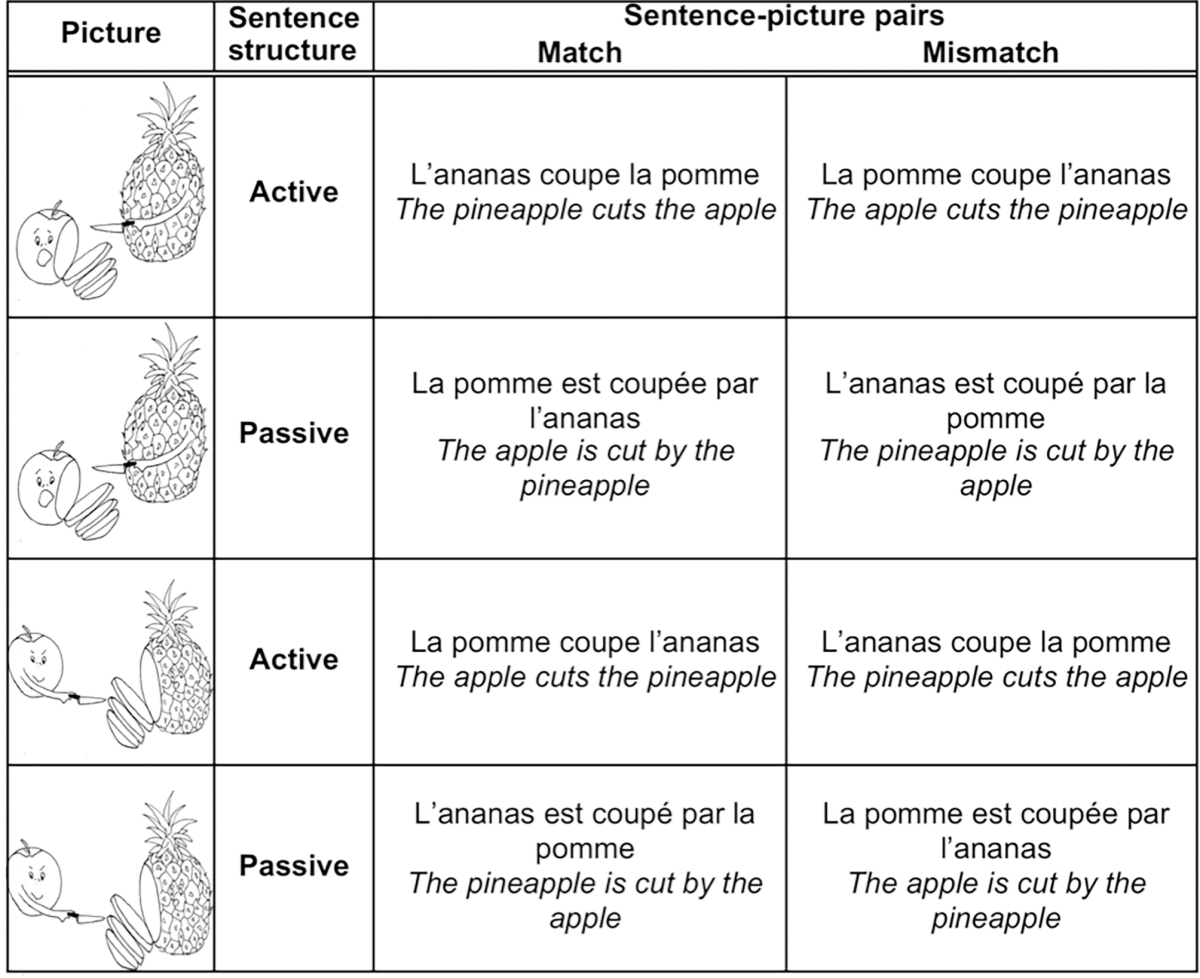
Sentence-picture pairs. Examples of Sentence-Picture pairs for four sentences with the same words.

Eight series of two characters and one verb (e.g. pineapple, apple, to cut) were created (Table 1). From each series, we created 4 sentences by inverting agent and patient, in active and passive voices. This resulted in 2 active and 2 passive sentences, which were pragmatically equivalent [45]. In total, we thus generated 16 active and 16 passive sentences (see S1 Table). Each sentence appeared half of the time paired with a picture matching the sentence and half of the time with a picture that inverted the agent and the patient (respectively “Match” and “Mismatch” in Fig 1). The location (right side or left side of the display) of the agent was counterbalanced across pictures.

**Table 1:** List of the materials used in the study.

French materials	English translation
ananas, couper, pomme	pineapple, to cut, apple
dauphin, peindre, pingouin	dolphin, to paint, penguin
girafe, doucher, zèbre	giraffe, to shower, zebra
raisin, photographier, citron	grape, to photograph, lemon
lion, mesurer, vache	lion, to measure, cow
carotte, arroser, fraise	carrot, to water, strawberry
banane, pêcher, poire	banana, to fish, pear
lune, parfumer, soleil	moon, to perfume, sun

We constructed two syntactically homogenous and two syntactically heterogeneous blocks [46]. In the homogeneous blocks, 16 active sentences or 16 passive sentences were repeated 8 times, resulting in 128 trials per block. In the heterogeneous blocks, 8 passive and 8 active sentences were repeated 8 times, also resulting in 128 trials per block. Homogeneous and heterogeneous blocks were alternated. Their order of presentation was counterbalanced across participants. The same pictures were used in homogeneous and heterogeneous blocks and for actives and passives.

Within the homogenous block, all trials shared the same syntactic structure (active or passive). The order of trials was pseudo-randomized such that no character or verb was repeated in two consecutive sentences.

The heterogeneous block contained the same number of active and passive sentences. For each participant, the order of presentation was pseudo-randomized to prevent participants from having any expectation about consecutive sentences. We did not allow sequences of more than three sentences sharing the same syntactic structure. Trials in which the preceding sentence shared the same lexical content were excluded from the analysis (2.6% of the trials).

**Fig 2:**
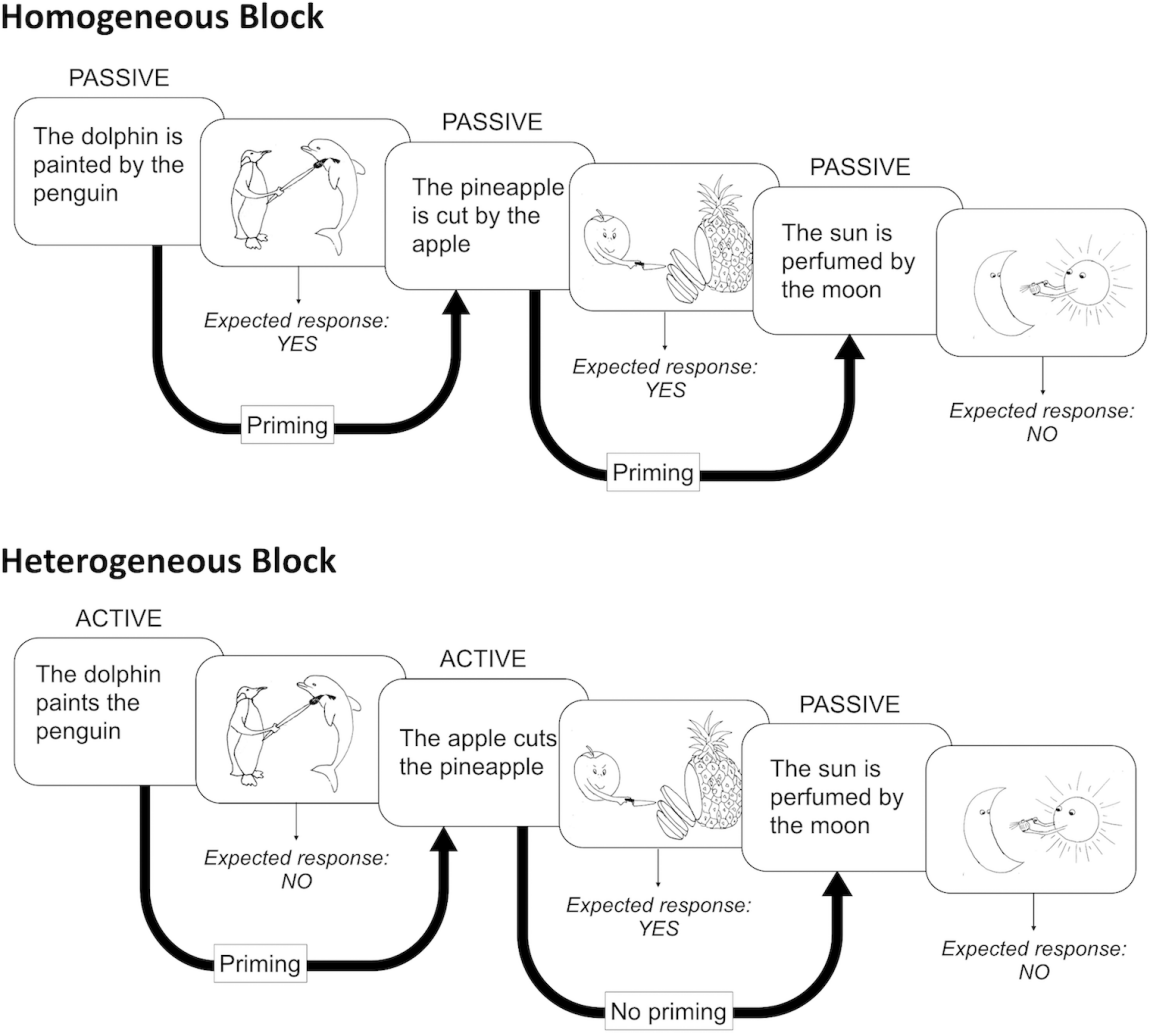
Structure of the homogenous and heterogeneous blocks used in the experiment. The top panel illustrates the structure of a homogeneous block, using the example of a passive block. The bottom panel illustrates the structure of a heterogeneous block, in which both active and passive sentences are presented. Thick arrows indicate whether or not priming is expected between two consecutive sentences. Expected responses on the sentence-picture pairs are also indicated.

Fig 2 illustrates the block structure used in the experiment.

### Procedure

The experiment was conducted in a quiet room on a laptop computer (15.4” LCD) using E-PRIME ®1.1.4.1 software. All sentences were recorded by a male native French speaker and digitized with Audacity™ GNU software. Participants were familiarized to the experiment with three practice trials (not included in the analysis). Each trial started with a fixation cross lasting 1000 ms in the center of the screen, followed by the presentation of the sentence through headphones. At the offset of the sentence the picture appeared and filled the screen until a response was given or until a time-off after 2800 ms. Participants were instructed to press the buttons of an external response box with their dominant hand, to answer whether the picture correctly represented the sentence (Yes or No response). Reaction times (RTs) were time-locked to the onset of the picture presentation. The inter-stimulus interval was 1000 ms. The experiment lasted approximately 50 min.

### Data analysis

Four analyses were conducted to test the effect of structural priming on participants’ performance. The first two analyses looked at participants’ performance in the homogeneous and in the heterogeneous condition, first looking at the effect of block and sentence type on performance, second looking at the effect of block type and trial number on participants’ speed of response. The third analysis looked within the heterogeneous block at the effect of being preceded by a sentence sharing the same structure (i.e. one repetition), as compared to having no structural overlap between consecutive sentences (i.e. no repetition). Finally, the third analysis looked at the effect of being preceded by two sentences sharing the same structure (i.e. two repetitions) as compared to one (i.e. one repetition). For all four analyses both accuracy and reaction times were analyzed with linear mixed effects models run in R with the package lme4 [47]. The dependent variable was either accuracy (correct or incorrect), analyzed with a binomial generalized linear mixed effects model, or the log 10 transform of reaction times, analyzed with a linear mixed effects model. For the reaction time analysis only correct responses were analyzed. Reaction times of 0 ms were removed to allow for the log transformation. All fixed effects were coded with contrast coding with the exception of trial number in the second analysis, which was coded as a continuous variable. For all models, we included by-participant and by-item random slopes and intercepts and kept the maximal random structure that converged [48]. Significance was assessed via model comparison with alpha set at 0.05.

## Results

### Homogeneous vs. heterogeneous condition

**Fig 3:**
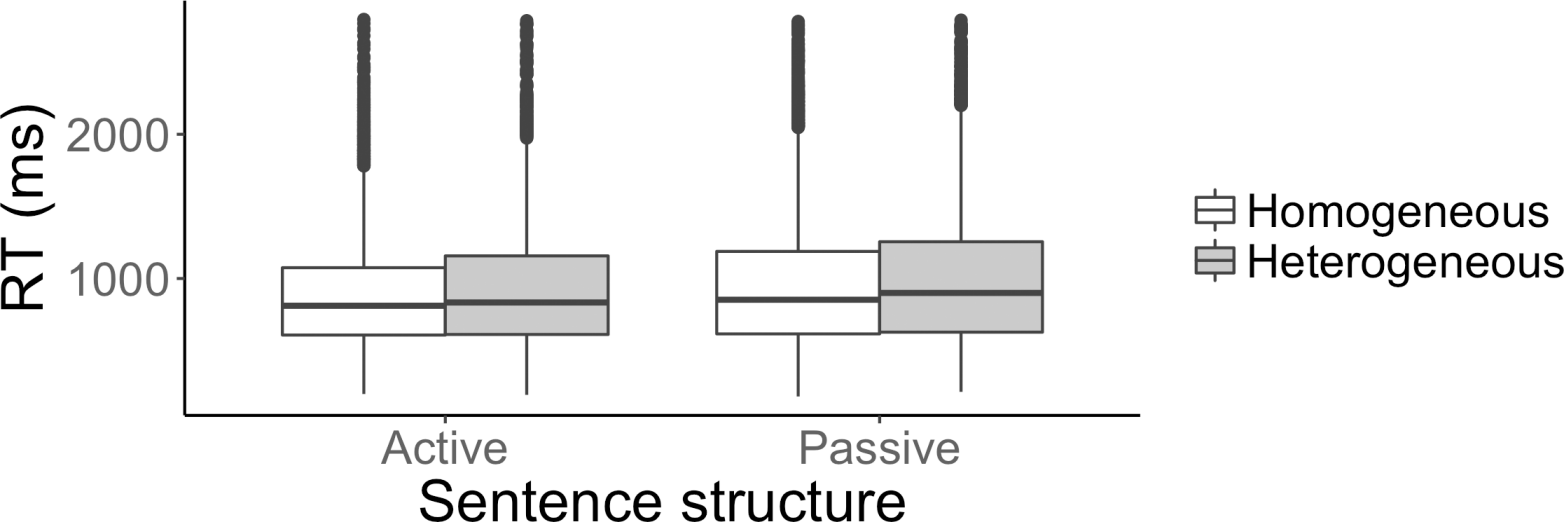
Homogeneous vs. heterogeneous condition. Reaction times (ms) for active and passive sentences in the homogeneous block (in white) and in the heterogeneous block (in grey).

The first analysis looked at the effect of block type (homogeneous, heterogeneous) on participants’ performance. The fixed effects were block type and sentence structure (active, passive). Reaction times are plotted in Fig 3 below.

For the accuracy analysis, the model that converged included item as a random intercept and participants both as a random intercept and random slope by sentence structure, correlated with the random intercept. There was a significant effect of block type, such that participants were less accurate in the heterogeneous than in the homogeneous block [β = -0.17, SE = 0.08, χ2(1) = 4.5, p < 0.05], and no effect of sentence structure. The interaction of block type and sentence structure was not significant. For the reaction time analysis, the model that converged included item as a random intercept and participant as both a random intercept and a random slope by block type, sentence structure and interaction of block type with sentence structure, correlated with the random intercept. There was a significant effect of block type, such that participants were faster in the homogeneous than in the heterogeneous block (respectively, mean 925ms (SE 31ms) and mean 963ms (SE 32ms)) [β = 0.02, SE = 0.007, χ2(1) = 4.02, p < 0.05], and a significant effect of sentence structure, such that participants were faster in active than in passive sentences (respectively, mean 910ms (SE 27ms) and mean 979ms (SE 35ms)) [β = 0.02, SE = 0.01, χ2(1) = 4.3, p < 0.05]. The interaction of block type and sentence structure was not significant.

### Homogeneous and heterogeneous condition: Effect of trial number

**Fig 4:**
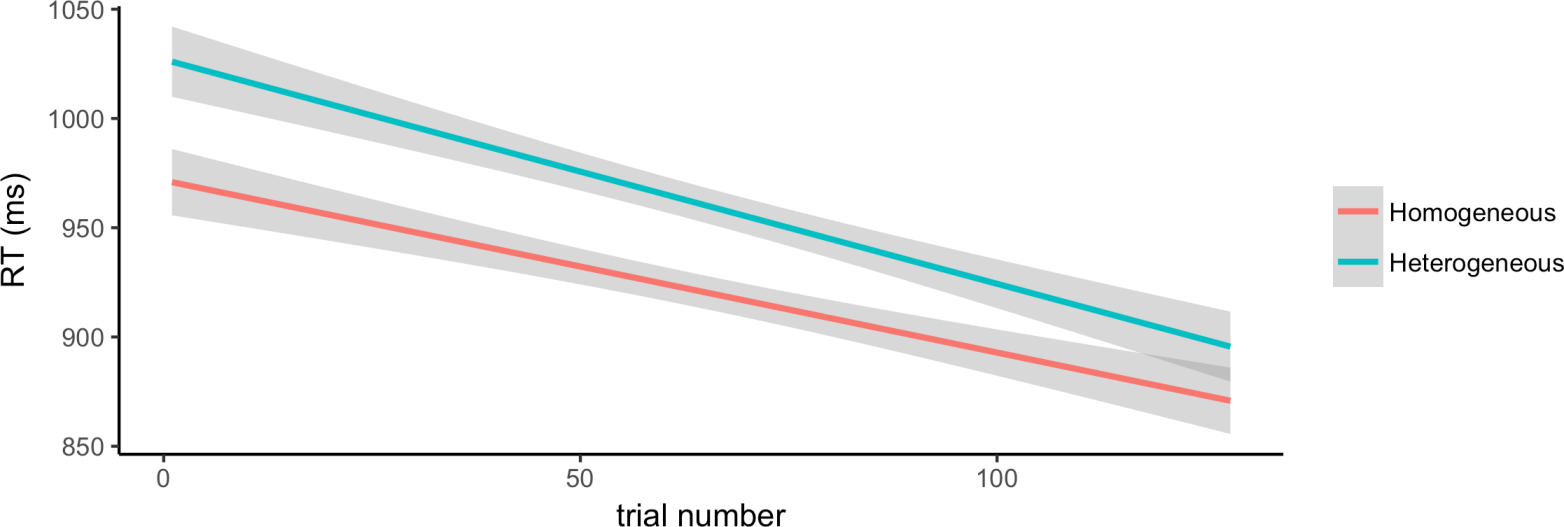
Effect of trial number on RT. Linear plot of participants’ reaction times (ms) as a function of trial number, by block type (heterogeneous and homogeneous).

The second analysis looked at the effect of trial number within each block on participants’ RTs. The fixed effects were trial number (1–128) and block type (homogeneous, heterogeneous). Reaction times are plotted in Fig 4 below.

The model that converged included item as a random intercept and participant both as a random intercept and a random slope by block type, correlated with the random intercept. There was a significant effect of block type, such that participants were faster in the homogeneous than in the heterogeneous block [β = 0.02, SE = 0.008, χ2(1) = 5.13, p < 0.05], and a significant effect of trial number within block, such that participants’ response speed increased as they progressed into a given block [β = -0.0004, SE = 2.86e-05, χ2(1) = 207.57, p < 0.001]. The interaction of block type and trial number was not significant.

### Heterogeneous condition: Effect of syntactic repetition

**Fig 5:**
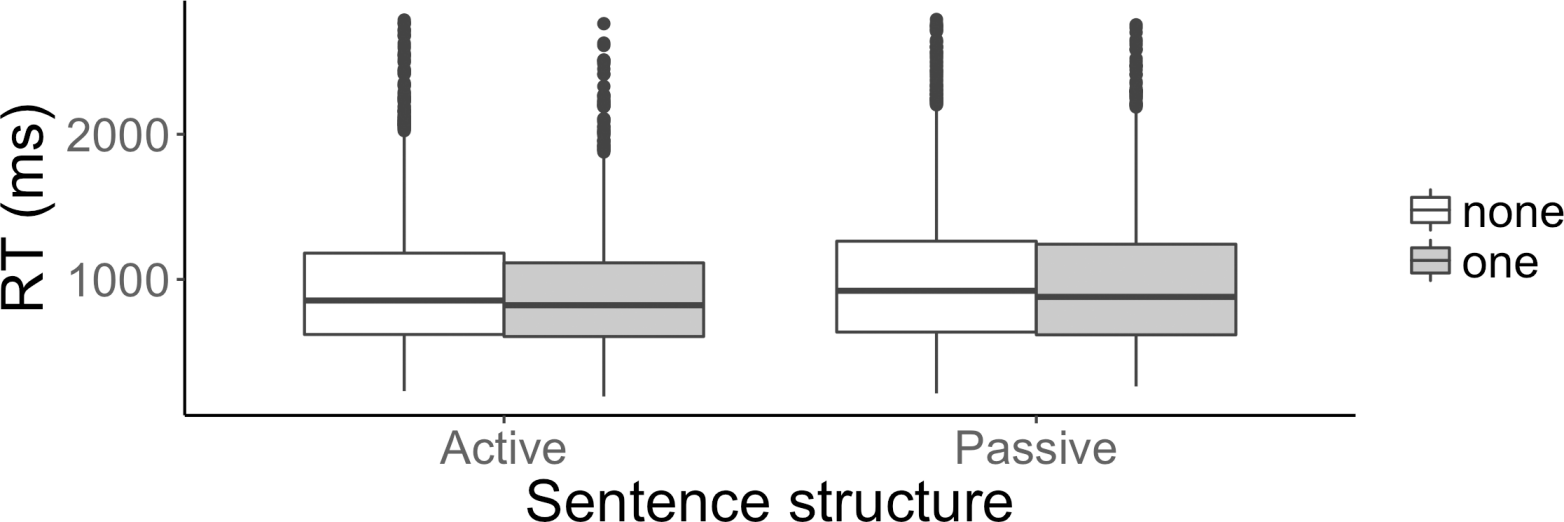
Effect of syntactic repetition within the heterogeneous condition. Reaction times (ms) for active and passive sentences preceded by one sentence sharing the same syntactic structure (in white) and by no sentence sharing the same syntactic structure (in grey).

The third analysis looked for an effect of structural priming within the heterogeneous condition, focusing on whether a given sentence was preceded or not by one sentence sharing the same syntactic structure without lexical repetition. The fixed effects were Repetition (none vs. one) and sentence structure (active, passive). Reaction times are plotted in Fig 5 below.

For the accuracy analysis, the model that converged included item as a random intercept and participant both as a random intercept and a random slope by repetition, uncorrelated with the random intercept. There was no effect of sentence structure, no effect of having one vs. no repetition, and no interaction of repetition and sentence structure. For the reaction time analysis, the model that converged included item as a random intercept and participant both as a random intercept and a random slope by repetition, sentence type and interaction of repetition with sentence type, correlated with the random intercept. There was a significant effect of repetition, such that participants were faster when the preceding sentence shared the same syntactic structure (respectively, mean 948ms (SE 33ms) and mean 978ms (SE 33ms)) [β = -0.01, SE = 0.003, χ2(1) = 15.5, p < 0.001], and a significant effect of sentence structure, such that participants were faster in active than in passive sentences (respectively, mean 939ms (SE 30ms) and mean 1001ms (SE 36ms)) [β = 0.02, SE = 0.01, χ2(1) = 3.9, p < 0.001]. The interaction of repetition and sentence structure was not significant.

### Heterogeneous condition: Effect of one versus two syntactic repetitions

The fourth analysis also looked within the heterogeneous condition, to assess whether increasing the number of sentences sharing the same syntactic structure affected amount of structural priming. Since there were never more than 3 sentences sharing of the same syntactic structure, this analysis looked at cases of one vs. two repetitions. The fixed effects were Repetition (one vs. two) and sentence structure (active, passive).

For the accuracy model, there was no effect of sentence structure, no effect of having one *vs*. two repetitions, and no interaction of repetition and sentence structure. For the reaction time analysis, the model that converged included item as a random intercept and participant both as a random intercept and a random slope by repetition and, sentence structure and interaction of repetition with sentence structure correlated with the random intercept. There was no effect of sentence structure, no effect of number of repetitions, i.e. participants were not faster when the two preceding sentences shared the same syntactic structure *vs* one, and no interaction of repetition and sentence structure.

## Discussion

This study introduces a new experimental paradigm to investigate structural priming in sentence comprehension. We used an outcome dependent response that allows to more directly compare these effects with those found in sentence production. This allows us to assess whether–like in production–structural priming is observable in sentence comprehension, independent of lexical repetition and of participants’ expectations. We designed a sentence-picture matching task that boosts abstract syntactic processing and compared two conditions: a homogeneous condition in which syntactic structure was predictable and a heterogeneous condition in which no expectation about sentence structure could be built. First, we replicate the structural priming effect when participants can build expectations about the distribution of syntactic structures. Second, we show that our measure is sensitive enough to detect structural priming in comprehension with a single prime trial, in the lack of predictive effects to which participants could adapt.

Participants were faster in the homogeneous condition compared to the heterogeneous condition. Although in each block participants became faster at responding as they advanced in the block trials, this task-related facilitation was comparable in the two conditions. In the homogeneous condition, the lexical content was never repeated between two consecutive sentences. This finding replicates previous studies showing that sentence comprehension is facilitated when the syntactic structure of sentences is repeated and confirms that structural priming can be observed independent of lexical repetition [35, 36, 49]. However, in the homogeneous condition, the facilitation effect of syntactic repetition could be attributed to participants’ expectations. As in a habituation paradigm, participants hearing several sentences with the same syntactic structure will implicitly expect the next sentence to be syntactically similar and will be facilitated by this repetition [50, 51]. In fact, in the homogeneous condition participants could match the pictures and the sentences by detecting that the first word was always the agent (active sentence) or the patient (passive sentence), thus bypassing syntactic parsing.

The heterogeneous condition however prevented participants from developing expectations about the following sentence and forced them to syntactically parse each sentence. We found that having only one preceding sentence sharing the same syntactic structure caused participants to respond faster on the target sentence. This result is in line with those studies using online measures and showing abstract structural priming in sentence comprehension independent of lexical repetition, even when participants cannot adapt their expectations about the distribution of syntactic structures [17, 32, 37, 38, 40, 41]

What remains to be explained is how these priming effects arise. Two mechanisms have been proposed to account for structural priming. Priming could arise from the transient activation of a structural representation [6, 23, 52], or be the result of an implicit learning mechanism [4, 50, 53–56]. According to the former mechanism, facilitation is attributed to the temporary activation of structural information stored in long-term memory and rapidly dissipates. In contrast, according to the implicit learning mechanism, adaptation is error-based, in that a mismatch between predicted and processed structure yields learning through adjustments in the representation system. Learning occurs as exposure increases: structure-building becomes easier as that structure is repeatedly built, giving rise to a more durable adaptation effect. Growing evidence supporting the implicit learning view suggests that during language processing listeners and speakers adapt their expectations about the distribution of syntactic structures to approximately match the statistics of their environment. Implicit learning thus takes place during language comprehension and language production, through the adaptations in the prediction errors experienced during the processing of a sentence. For instance, although a processing difficulty is associated with the processing temporally ambiguous sentences (e.g. “The woman heard the dog had barked all night”), the difficulty is dependent on how expected a given interpretation of the sentence is in a given environment [57, 58]. Structural priming would thus be a form of implicit learning arising through the repeated structure building experience [4, 59, 60]. This learning mechanism can account for the fact that structural priming may be durable, a fact which transient activation accounts fail to explain since changes in the activation of stored representations take place over a short timescale (see [4, 50, 61, 62]).

Priming effects in production studies are systematically reported even after a single prime trial. In previous comprehension studies however, these facilitatory effects on the target sentence are often induced by several primes, and only more rarely are they found after single primes (e.g. [35, 49]). In the heterogeneous condition of the present experiment, participants were forced to syntactically parse each sentence and they were prevented from building expectations about the structure of the upcoming sentence. Our results show that participants are faster when a sentence is preceded by only one sentence sharing the same syntactic structure. This priming effect could arise from one of the two underlying mechanisms outlined above. First, it could arise from the transient activation of a stored structural representation, in which case we would expect priming not to accumulate over time. In the present study, we do not observe an increase in priming as the number of primes increases (one vs. two repetitions). Second, it could arise from implicit learning, with priming accumulating over subsequent exposures. The absence of an accumulative effect is not due to a floor effect in the reaction times, as reaction times were significantly smaller in the homogeneous condition. Priming in our study is thus compatible with the first mechanism. However, since this study was not designed to directly distinguish between the two sources of priming, we cannot rule out a small accumulating effect, which may not be detectable with our paradigm.

Overall, this result sheds new light on the frequent lack of structural priming observed in many sentence comprehension studies. These previous studies used online measures such as reading-times and eye-tracking measures, and often contained ambiguous sentences producing garden-paths which may hide the priming effects [9]. On the contrary, the sentences we used only allowed for a single reading, which simplifies syntactic processing making potential priming effects more detectable. In addition, our paradigm (1) investigates structural priming in comprehension using an outcome dependent variable, as in production studies and (2) promotes the processing of abstract syntactic structure and its reuse across consecutive trials, since all sentences were reversible and pragmatically non-canonical, removing word order and world knowledge cues. Our results show that when participants are fully engaged in syntactic processing for sentence comprehension, we observe a structural priming effect. These data support that the degree to which listeners are engaged in syntactic analysis in sentence comprehension is a function of the syntactic demands of the task [23, 42, 43]. In previous studies that did not report a priming effect, participants could perform the task without fully parsing the sentence they read or listen to [12, 44].

Alternatively, structural priming might be present in sentence comprehension as in sentence production, but be undetectable by online tasks such as reading time and eye tracking. Indeed, these tasks may capture local and smaller units than those computed when the full sentence is processed, thus limiting the possibility of detecting the effect of structural priming [9, 40]. Such results are predicted by constraint-based models of sentence processing which propose that, during comprehension, all possible interpretations of the sentence are examined in parallel until the end of the sentence is reached and the lexical items are mapped to a definitive syntactic structure (Expectation based account: [63, 64]; Lexicalist account [20, 65]; Optimality theory [66]; Parallel architecture [42]). Accordingly, because processing of local syntactic structure is lexically tied [20], repetition of lexical items induces structural priming in online tasks [9]. Conversely, offline tasks tap full sentence processing when the analysis of the entire syntactic structure is accomplished and enhance the effect of abstract syntactic structure repetition.

The structural priming effect observed in the present study suggests that during comprehension a syntactic structure represented at an abstract level, i.e. independently of lexical content, facilitates the processing of subsequent sentences with the same syntactic structure. Both syntactic and lexical representations play a role in sentence comprehension. Indeed in sentence production and comprehension, there is evidence that structural priming effects are amplified when the lexical content is also repeated due to the “lexical boost” [6, 24, 67–69]. Our data however show that, like in production, structural priming in comprehension can be found independent of lexical repetition.

To conclude, when participants are required to perform full parsing, comprehension of subsequent sentences is facilitated when they share the same syntactic structure. In line with some previous studies, which found priming in comprehension, we detect structural priming independent of participants’ expectations by using an outcome-based dependent variable, with only one structural repetition sufficient to drive this effect. We thereby show that during sentence comprehension abstract syntactic structure may be processed independent of lexical content, just like in sentence production. Our data is thus consistent with the view that the source of structural priming in the two modalities lies in a similar–if not in the same–mechanism.

## Supporting information

S1 Table Experimental material. List of the sentences used in the experiment.
(PDF)
