# Supplementary material for: Structural priming in sentence comprehension: A single prime is enough
Source: PLoS One. 2018 Apr 2;13(4):e0194959. doi: 10.1371/journal.pone.0194959 (PMC5880384; doi:10.1371/journal.pone.0194959)
Supplement: S1 Table — List of the sentences used in the experiment. (PDF) [file pone.0194959.s001.pdf]

S1 Table Experimental material. List of the sentences used in the experiment

Active form	Passive form
La pomme coupe l'ananas	L'ananas est coupé par la pomme
The apple cuts the pineapple	*The pineapple is cut by the apple*
L'ananas coupe la pomme	La pomme est coupée par l'ananas
The pineapple cuts the apple	*The apple is cut by the pineapple*
La banane pêche la poire	La poire est pêchée par la banane
The banana fishes the pear	*The pear is fished by the banana*
La poire pêche la banane	La banane est pêchée par la poire
The pear fishes the banana	*The banana is fished by the pear*
La carotte arrose la fraise	La fraise est arrosée par la carotte
The carrot waters the strawberry	*The strawberry is watered by the carrot*
La fraise arrose la carotte	La carotte est arrosée par la fraise
The strawberry waters the carrot	*The carrot is watered by the strawberry*
Le raisin photographie le citron	Le raisin est photographié par le citron
The grapes photographs the lemon	*The grapes are photographed by the lemon*
Le citron photographie le raisin	Le raisin est photographié par le citron
The lemon photographs the grapes	*The grapes is photographed by the lemon*
La vache mesure le lion	Le lion est mesuré par la vache
The cow measures the lion	*The lion is measured by the cow*
Le lion mesure la vache	La vache est mesurée par le lion
The lion measures the cow	*The cow is measured by the lion*
La lune parfume le soleil	Le soleil est parfumé par la lune
The moon perfumes the sun	*The sun is perfumed by the moon*
Le soleil mesure la lune	La lune est parfumée par le soleil
The sun perfumes the moon	*The moon is perfumed by the sun*
Le pingouin peint le dauphin	Le dauphin est peint par le pingouin
The penguin paints the dolphin	*The dolphin is painted by the penguin*
Le dauphin peint le pingouin	Le pingouin est peint par le dauphin
The dolphin paints the penguin	*The penguin is pained by the dolphin*
Le zèbre douche la girafe	La girafe est douchée par le zèbre
The zebra showers the giraffe	*The giraffe is showered by the zebra*
La girafe douche le zèbre	Le zèbre est douché par la girafe
The giraffe showers the zebra	*The zebra is showered by the giraffe*
